# Use of Sequenom Sample ID Plus® SNP Genotyping in Identification of FFPE Tumor Samples

**DOI:** 10.1371/journal.pone.0088163

**Published:** 2014-02-13

**Authors:** Jessica K. Miller, Nicholas Buchner, Lee Timms, Shirley Tam, Xuemei Luo, Andrew M. K. Brown, Danielle Pasternack, Robert G. Bristow, Michael Fraser, Paul C. Boutros, John D. McPherson

**Affiliations:** 1 Department of Genome Technologies, Ontario Institute for Cancer Research, Toronto, Canada; 2 Ontario Cancer Institute/Princess Margaret Hospital, Toronto, Canada; 3 Informatics & Biocomputing Platform, Ontario Institute for Cancer Research, Toronto, Canada; 4 Department of Medical Biophysics, University of Toronto, Toronto, Canada; 5 Department of Pharmacology & Toxicology, University of Toronto, Toronto, Canada; University College London, United Kingdom

## Abstract

Short tandem repeat (STR) analysis, such as the AmpFlSTR® Identifiler® Plus kit, is a standard, PCR-based human genotyping method used in the field of forensics. Misidentification of cell line and tissue DNA can be costly if not detected early; therefore it is necessary to have quality control measures such as STR profiling in place. A major issue in large-scale research studies involving archival formalin-fixed paraffin embedded (FFPE) tissues is that varying levels of DNA degradation can result in failure to correctly identify samples using STR genotyping. PCR amplification of STRs of several hundred base pairs is not always possible when DNA is degraded. The Sample ID Plus® panel from Sequenom allows for human DNA identification and authentication using SNP genotyping. In comparison to lengthy STR amplicons, this multiplexing PCR assay requires amplification of only 76–139 base pairs, and utilizes 47 SNPs to discriminate between individual samples. In this study, we evaluated both STR and SNP genotyping methods of sample identification, with a focus on paired FFPE tumor/normal DNA samples intended for next-generation sequencing (NGS). The ability to successfully validate the identity of FFPE samples can enable cost savings by reducing rework.

## Introduction

STR profiling is a powerful, multiplex PCR-based assay that uses up to 16 tetranucleotide loci repeats [1], [2]. Generally, the certainty of identity with one locus is less than 0.01, and is 1.0×10^−15^ for 12 or more loci, making STR a very effective method for sample identification [2], [3]. Despite its efficacy, improvements to STR assays have been required for forensic analysis on highly-degraded DNA samples, namely reducing amplicon size to increase amplification efficiency [4], [5]. Previous studies on degraded DNA have demonstrated that STR analysis using amplicons of 280 nt or less generates 48% more genotype profiles than would be possible with longer STRs [5]. STR analysis has been advocated by the American Type Culture Collection Standards Development Organization (ATCC SDO) Workgroup for human cell line authentication in general [6], [7]. Monitoring sample quality at the beginning of a process is especially essential to ensure that cell lines and tissues are authenticated for correct input DNA in order to ensure validity of data output [8]. Failure to authenticate samples may result in unwanted, incorrect, or duplicate data; consequently, these superfluous errors can be costly. Common causes leading to sample misidentification include mislabeling of cell cultures or DNA samples, cross-contamination, co-cultivation, and xenograft propagation. For example, it has been revealed that at least 360 cell lines are known to have some cross-contamination [9]. Furthermore, STR profiling demonstrated the misidentification of multiple ovarian cancer cell lines [10]. Formalin fixed paraffin embedded (FFPE) sample misidentification is likely to occur from some of the same types of errors and circumstances. Next generation sequencing (NGS) technology has rapidly progressed and expanded over the past decade [11], [12]. With such abrupt growth and development, there is an increasing need for accuracy at each stage of a sequencing pipeline. Quality control checkpoints are typically implemented throughout the process, including STR profiling which is now regularly used to verify sample identity after sample reception, or before NGS library preparation. Furthermore, with NGS technology now capable of producing whole genome and exome data from FFPE-derived DNA, it will be beneficial for 100% of tissue authentication to be successful despite inputs of fragmented DNA. In a clinical setting, FFPE tissues are an indispensable DNA resource and the standard for pathological examination of tissues; however, this preservation method is typically prone to analyte degradation [13]. To accommodate such DNA size limitations for NGS sample quality control, a further reduction in amplicon size is necessary. A single nucleotide polymorphism (SNP) genotyping panel has been designed to require amplification of shorter targets, and provide greater than 1×10^−18^ discriminatory power [14], [15]. This study explores the use of Sample ID Plus® SNP assay technology (Sequenom Bioscience, San Diego, CA) in generating complete genotype profiles from FFPE tissue DNA.

## Materials and Methods

### Ethics Statement

FFPE samples were taken as part of a larger NGS study of genomic prognostic factors within pre-treatment biopsies derived from prostate cancer patients with Institutional Review Board (IRB) approval and patient written consent (Canadian Prostate Cancer Genome Network (CPC-GENE) project; University Health Network-Research Ethics Board UHN06-0822-CE and UHN11-0024CE [16]. The samples analyzed in this study were taken from a larger deep-sequencing dataset deposited in the European Genome-phenome Archive (dataset ID EGAS00001000549).

### Sample Extraction

DNA was extracted from patient blood samples (N1-2, N2-2, N3-2, AND N4-2) using the ArchivePure DNA Blood Kit from 5 Prime (Gaithersburg, MD) following the manufacturer’s recommended protocol. DNA was also extracted from corresponding FFPE prostate tumors (T1-2, T2-2, T3S1-2/T3S2-2, and T4-2). Two different sections of tumor sample 3, T3S1-2 and T3S2-2, were extracted and tested as individual samples, but were both paired with N3-2. The approximate age of the FFPE tissue blocks were as follows: T1-2, 5 years; T2-2 and T4-2, 8 years; T3S1-2 and T3S2-2, 11 years. FFPE prostate tumors were macro-dissected, proteinase K-treated, and phenol-chloroform extracted using a modified protocol based on the xylene-free MagMAX FFPE DNA Isolation Kit (Life Technologies, Carlsbad, CA).

### STR Genotyping

For STR profiling with the AmpFlSTR® Identifiler® Plus PCR Amplification Kit (Applied Biosystems, Foster City, CA), 25 ng of FFPE tumor/normal DNA or 5 ng of R1 reference DNA was used. Data were analyzed using GeneMarker HID V1.95. The mean size of the 15 STR markers evaluated in this study range from 101–359 nt; the amelogenin sex determination marker was excluded from analysis for simplicity since gender information was not required. STR marker sizes are displayed as calculated fractions based on comparisons to internal size standards performed by Applied Biosystems. Individual markers have variable, but specific size ranges.

### Multiplex PCR FFPE DNA Quality Assessment

For multiplexed PCR, four primer pairs (IDT) were used to amplify 100, 200, 300, and 400 bp non-overlapping regions of the GAPDH gene from 5 ng of each sample [17]. The products were amplified in a 50 µl reaction, the final concentrations of 100 F/R and 300 F/R were 0.133 µM, 200 F/R were 0.200 µM, and 400 F/R were 0.067 µM. Multiplex PCR reaction conditions were as follows: 98°C for 30 sec, followed by 35 cycles each of 98°C for 10 sec, 62°C for 30 sec, and 72°C for 30 sec with a final 10 min extension at 72°C. PCR was carried out using 1 U Phusion HF *Taq* DNA polymerase and buffer (NEB, Ipswitch, MA), 500 mM KCl, and 10 mM dNTPs (Invitrogen, Carlsbad, CA). For positive controls, the same PCR reaction was performed on corresponding normal samples, as well as on a reference prostate gDNA, R1. A no template control (NTC) consisting of nuclease-free water (Ambion, Carlsbad, CA) was also included. All PCR reactions were purified using the MinElute PCR Cleanup Kit (QIAGEN, Venlo, Netherlands), and quantified with a Qubit 1.0 fluorimeter (Invitrogen). To evaluate GAPDH amplification, gel electrophoresis was performed on PCR reactions using a 2% agarose gel. PCR product sizes and quantity of approximately 100, 200, 300, 400 bp were confirmed with a DNA Bioanalyzer assay (Agilent Technologies, Santa Clara, CA).

### SNP Genotyping

For Sequenom Bioscience’s Sample ID Plus® panel, 10 ng of FFPE tumor/normal DNA or 2.5 ng of R1 reference DNA was used to determine 47 SNP calls; however a total of 45 SNPs were evaluated due to the general failure of two SNP calls, rs735155 and rs1029047. These two SNP calls also failed consistently in previous, unrelated assay tests; therefore, they were excluded from our analysis. In addition, other studies have observed similar poor performances for rs735155 and rs1029047 [18]. This method consists of five steps: PCR amplification, shrimp alkaline phosphatase treatment, single base extension, nanodispensing, and matrix-assisted laser desorption/ionization time of flight (MALDI-TOF) mass spectrometry [19]. Two previously confirmed primary non-FFPE tumor/normal pair matches (Pos1-T, Pos1-N and Pos2-T, and Pos2-N) were used as positive controls. Two mouse samples, Neg1 and Neg2, were used as negative controls. NTC wells were also included on the assay plate. All SNP calling from Sequenom Typer v4.0.20 software reports was analyzed using Microsoft Excel 2010 spreadsheets. Sex determination markers were noted but excluded from analysis to retain consistency with STR profiling analysis. Current SNP amplicon sizes ranged from 76–139 bp. Heat map analysis plots were generated by R v2.15.1.

### DNA Fragmentation

To simulate limited DNA size as observed in FFPE samples, 3 µg of reference gDNA, R1, was fragmented by sonication using a Covaris S2 instrument. Performance of a shearing time-course determined that 75 sec was an appropriate shearing time for this sample in order to obtain a broad fragment range when standard 400 bp DNA shear conditions were applied as follows: 10% duty cycle, intensity 4, 200 cycles per burst, 6–8°C water bath. The R1 shearing profile was confirmed with a DNA Bioanalyzer assay, followed by electrophoresis with a 2% agarose gel. From this gel, size selection was done manually for targeted R1 fragment pools of approximately 100, 200, 300, and 400 bp. These sizes were selected to coincide with GAPDH target regions used for prior FFPE DNA quality assessment. Gel cuts were purified with Zymoclean Gel DNA Recovery columns (Zymo Research, Irvine, CA). Purified R1 pools were quantified with a Qubit 1.0 fluorimeter (Invitrogen). Using a DNA Bioanalyzer assay, actual size-selected R1 targets were detected as 132, 233, 347, and 450 bp, and fragment pools were correspondingly named R1 132, R1 233, R1 347, and R1 450.

## Results and Discussion

**Figure 1:**
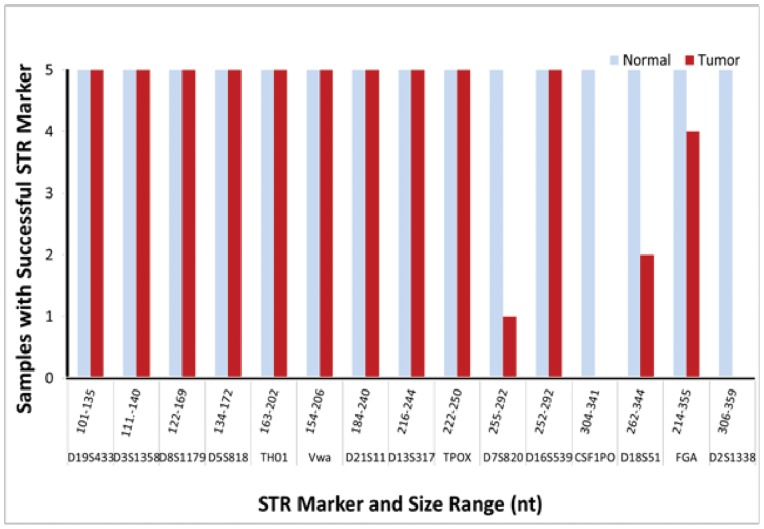
Frequency of STR marker detection. T1-2/N1-2, T2-2/N2-2, T3S1-2/T3S2-2/N3-2, T4-2/N4-2 tumor and normal samples are represented by bars only when an STR marker was successfully amplified. Nucleotide size ranges are listed above the corresponding STR marker.

To confirm FFPE sample identification, STR profiling was performed. Typically, at least 80% or more of alleles are required to match in order to properly authenticate DNA samples using STR profiling [20]. However, recent examinations indicate that, depending on the sample type, even more stringent thresholds would be beneficial for validating sample identity [21]. For sensitive and costly downstream applications such as NGS, at least 85% concordance was more reliable. Profiles were successfully obtained for all normal samples; however, several STRs consistently failed for each tumor sample (Figure 1). The failed STR markers ranged from 255–359 nt. Except for the success of the 274 nt D16S539 marker, data suggest that failure to generate a complete profile for FFPE samples was likely due to fragmentation and degradation of DNA impeding amplification of the larger amplicons. To predict successful array comparative genomic hybridization (aCGH) with FFPE DNA, van Beers *et al*. established a reliable multiplexed PCR assay which targeted 100, 200, 300, and 400 bp non-overlapping regions of the GAPDH gene [17]. We applied this assay to our FFPE tumor/normal pairs to explore the effects of DNA fragment size on successful amplification. Although all GAPDH regions amplified for all normal samples, PCR products were not seen for all four of the targeted regions for the corresponding tumor samples (Figure S1). The inability to amplify the larger GAPDH targets in FFPE samples implied that fragmentation of DNA could affect detection of loci greater than 200 bp in length. However, this result is not exhaustive, as formalin fixation can alter the DNA is other ways which could contribute to whether or not detection is possible [22], [23].

**Figure 2:**
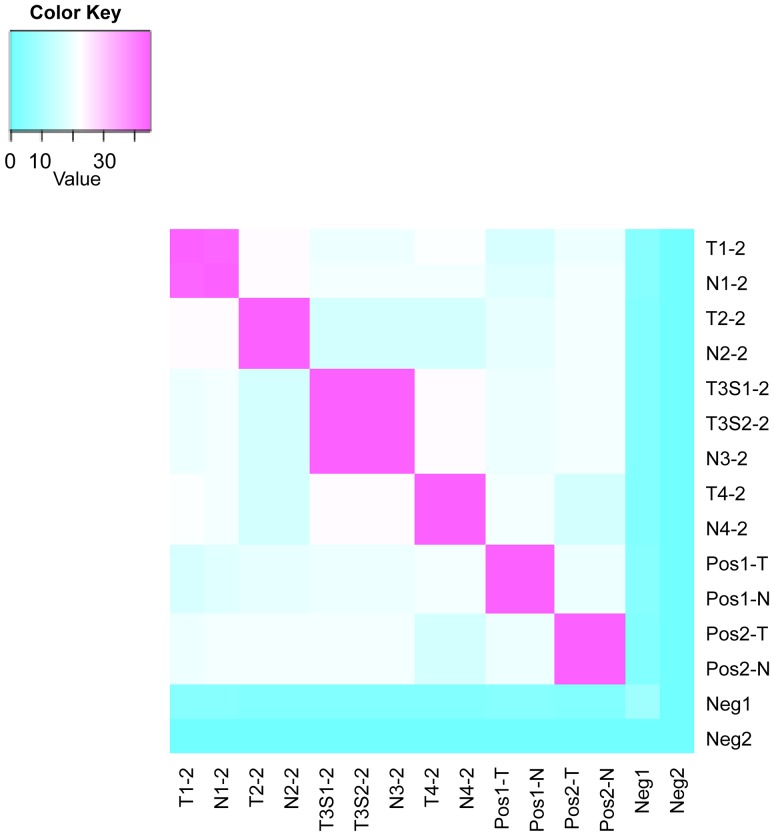
FFPE tumor/normal pair relatedness assessed by Sequenom SNP genotyping. Heat map analysis plots generated by R v2.15.1 visualize the number of matched SNP calls between paired samples. Positive (Pos1-T/N and Pos2-T/N) and negative (Neg1 and Neg2) controls are included in each heat map. Match failures are considered to be in the range of 0–38 SNP matches, while matched samples (38–45 SNP matches) are shown in pink.

To evaluate the capability of SNP genotyping to confirm the identities of the FFPE tumor/normal pairs subjected to STR profiling, samples were processed with Sequenom Bioscience’s Sample ID Plus® panel. The average number of matched SNP calls between unrelated samples in previously-developed identification panels has been described as 40.38%, and ranging from 13.46–78.85% [14]. Additionally, Gilbert *et al.* observed a 86–100% matched call rate on relatively higher quality FFPE samples when utilizing a similar 44-SNP genotyping method [24]. According to Sequenom Bioscience, samples are called mismatches when less than 30 SNP calls are concordant. However, to be consistent with the concordance rate of 85% we used for STR profiling, at least 38 SNP matches were required in our study. Heat map analysis illustrated that all FFPE tumor/normal pairs were detected (Figure 2). The T1-2/N1-2 pair displayed a 97.78% (44/45) call rate, as the rs104954 locus did not match. All other tumor/normal pairs and positive control pairs obtained 100% call rates. In addition, all other possible sample-sample, control-control, and sample-control combinations showed 0–51.11% (0–23) matched SNP calls, indicating no other relationships existed. Overall, successful SNP genotyping profiles were obtainable for FFPE tumor DNA, despite incomplete STR profiles. Furthermore, STR loci are more likely to have greater genetic instability than biallelic SNPs [25]. This and other factors, such as relatedness, could affect the minimal match threshold of an FFPE tumor/normal pair. However, the high success rate of SNP genotyping in this case renders these factors insignificant when compared to STR profiling results. These data demonstrated the efficacy and sensitivity of the Sequenom Bioscience’s Sample ID Plus® SNP genotyping panel over typical STR profiling methods, especially in the case of FFPE or types of highly-degraded samples.

**Figure 3:**
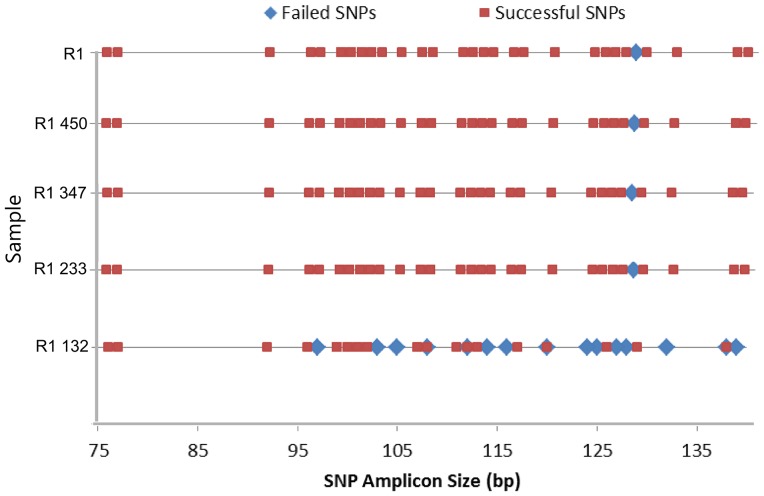
SNP amplicon size does not indicate SNP call failure. All 45–139 bp. One SNP amplicon of 128 bp (dbSNP ID rs733164) does not amplify in all instances. For R1 132, amplification fails for 14 additional SNPs ranging in size from 97–139 bp.

To further support that SNP genotyping, compared to STR profiling, is largely unaffected by target DNA size we devised a three-step approach: shear a reference DNA sample and separate into fragment pools, submit pools to STR profiling, and perform a SNP genotyping assay on the same pools. STR genotyping was performed on each sheared R1 fragment pool, and on intact R1 gDNA using the same methods described for the FFPE tumor/normal pairs (Table 1). All STR markers amplified successfully for R1 450, R1, C1, and C2. Six STR markers ranging from 216–359 nt expectedly failed for R1 132. Additionally, the larger TH01, vWA, D21S11, TPOX, and FGA STR markers were profiled successfully for R1 132. Bioanalyzer traces revealed that trace amounts of larger DNA fragments were present in the R1 132 fragment pool, which is likely why these unexpected STR markers amplified. All other STR marker failures were consistent with the R1 fragment pool size cutoffs, with the exception of CSF1PO, which failed for R1 347 despite its average size of 324 nt.. It was important to determine if SNP genotyping could be more effective at generating complete identification profiles than STR profiling using samples of known fragment size. For each sample, Figure 3 displays all SNP amplicons in the assay based on size, and classifies each as failed or successful. One SNP (dbSNP ID rs733164) failed to amplify for R1 and all fragment pools, despite its amplicon length of 128 bp. All other 44 SNP amplicons were successfully called for R1, R1 233, R1 347, and R1 450, providing a 97.78% call rate. In contrast, only 66.67% (30/45) of SNP calls were detected for R1 132. To reach the STR profiling concordance rate of 85%, at least 38 SNP calls are required to match. For all 15 failed SNP calls for R1 132, the amplicon sizes ranged from 97–139 bp, while the sizes varied from 76–138 bp for the successful SNP amplicons. Therefore, there appeared to be no direct relationship between amplicon size and a SNP’s successful detection. However, 97.78% (44/45) of SNP calls were detected when approximately twice the amount (5 ng) of R1 132 DNA input was utilized for genotyping, suggesting that the assay may be more sensitive to sample input quantity when DNA is fragmented due to the lower amounts of intact amplicon target. Johansen *et al.* described the impact of low DNA input amounts on allelic dropouts as well [18]. Overall, these fragment pool results support that DNA fragment integrity can limit STR profiling success, whereas DNA sample quantity, but not amplicon size, affects SNP genotyping results.

**Table 1:** STR marker amplification for R1 fragment pools and intact R1 DNA.

STR Marker	Amplicon Size (nt)	R1 132	R1 233	R1 347	R1 450	R1	C1	C2
D19S433	101–135	Y	Y	Y	Y	Y	Y	Y
D3S1358	111–140	Y	Y	Y	Y	Y	Y	Y
D8S1179	122–169	Y	Y	Y	Y	Y	Y	Y
D5S818	134–172	Y	Y	Y	Y	Y	Y	Y
TH01	163–202	Y	Y	Y	Y	Y	Y	Y
vWA	154–206	Y	Y	Y	Y	Y	Y	Y
D21S11	184–240	Y	Y	Y	Y	Y	Y	Y
D13S317	216–244	N	Y	Y	Y	Y	Y	Y
TPOX	222–250	Y	Y	Y	Y	Y	Y	Y
D7S820	255–292	N	N	Y	Y	Y	Y	Y
D16S539	252–292	N	N	Y	Y	Y	Y	Y
CSF1PO	304–341	N	N	N	Y	Y	Y	Y
D18S51	262–344	N	N	Y	Y	Y	Y	Y
FGA	214–355	Y	Y	Y	Y	Y	Y	Y
D2S1338	306–359	N	N	Y	Y	Y	Y	Y

SNP genotyping analysis was successful for FFPE tumors and the R1 132 and R1 233 fragment pools, where STR profiling efforts were rendered incomplete. Table 2 summarizes the input and results comparison between each genotyping method. Extraction method and sample age are variables that may have contributed to poor FFPE DNA quality, and subsequent STR failure. A method recently developed to extract DNA from FFPE tissue by xylene-free deparaffinization was directly compared to the QIAamp DNA FFPE Tissue Kit (QIAGEN), and showed that although greater yields were recovered, the DNA integrity suffered [26]. Another study showed that application of an automated, xylene-free deparaffinization approach allowed for 99% of samples to be analyzed by SNP genotyping, which yielded higher than 91% SNP calls [27]. In our study, FFPE tissues were not deparaffinized using xylene; instead the proteinase K incubation time was extended to last overnight at 55°C to maximize DNA recovery. The age of FFPE tissue blocks has also been shown to negatively affect DNA quality and quantity, especially after 5–6 years of storage [28]. For our study, all FFPE tumor DNA was extracted from blocks ranging in age from 5–11 years. Age alone is likely not the single factor here but rather changes in formalin fixation protocols in the intervening years. Comparative DNA extraction method studies employing STR analysis on FFPE tissues of variable age have indicated that increased storage time, extraction method, and reagent quality all play key roles in poor DNA quality [23]. Additionally, the aforementioned study demonstrated that decreasing amplicon size is most appropriate for retrieving improved results overall.

**Table 2:** Comparison of STR and Sample ID (SID) Plus genotyping methods used for FFPE samples.

	STR Genotyping	SID Genotyping
Number of Loci Used	15	45
DNA Input (ng)	25	10
Amplicon Size Range	101–359 (median = 219) nt	76–139 (median = 107) bp
Failed Amplicon Size Range	255–359 (median = 298) nt	97–139 (median = 120) bp
Lowest % Matched Calls Observed	73.33	97.78

## Conclusions

Altogether, the data presented in this report support the efficacy of Sequenom Bioscience’s Sample ID Plus® SNP assay technology in generating genotype profiles from FFPE tissue DNA to achieve accurate sample authentication for NGS application. The high genotyping success rate of this assay provided more definitive tumor/normal pair match results for FFPE samples than the corresponding STR profiles. It has been observed that SNP genotyping with this panel may not be adequate for forensic analysis due to low DNA input requirements and a lack of sensitivity [18]. However, this SNP genotyping platform is a promising and suitable alternative to STR profiling for NGS sample identification, especially for FFPE tissue DNA of lower integrity. In the future, application of the Sample ID Plus® panel as a quality control checkpoint before NGS library preparation will add cost savings by reducing rework due to sample mismatches. Furthermore, the ability to utilize FFPE-derived retrospective DNA samples for genomic analyses will be extremely important in cancers such as prostate cancer where the long natural history of the disease limits prospective assessments.

## Supporting Information

Figure S1 **Multiplex PCR amplification of target GAPDH regions.** Tumor samples (T1-2, T2-2, T3S1-2, T3S2-2, and T4-2) display failed or lower amplification of larger targets. Corresponding normal samples (N1-2, N2-2, N3-2, N4-2) are shown for comparison. A no template control (NTC) and a positive reference control (R1 (Pos)) lane are shown.
(TIF)
