# Supplementary figures and images for: Use of Sequenom Sample ID Plus® SNP Genotyping in Identification of FFPE Tumor Samples
Source: PLoS One. 2014 Feb 13;9(2):e88163. doi: 10.1371/journal.pone.0088163 (PMC3923782; doi:10.1371/journal.pone.0088163)

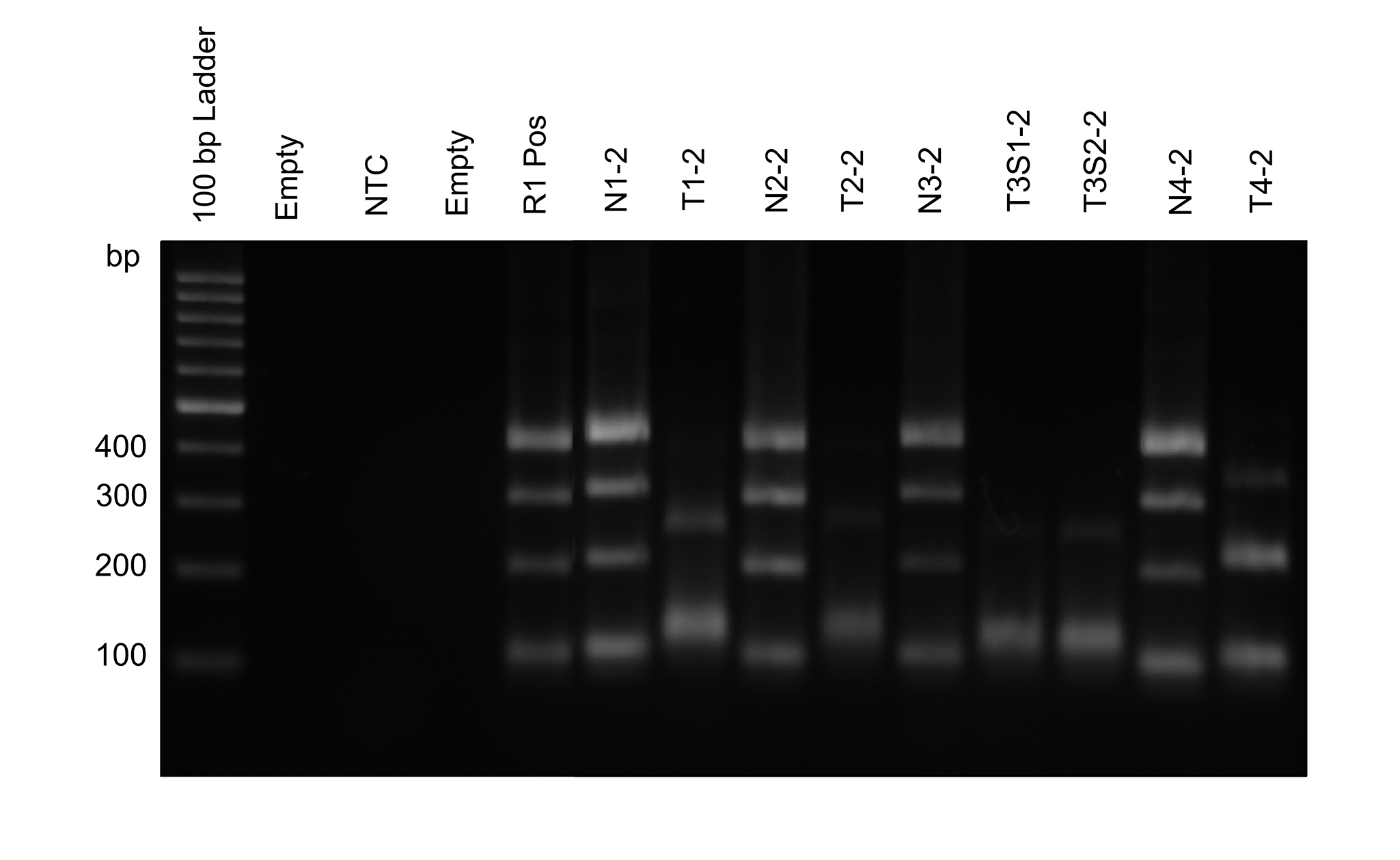

Supplement: Figure S1 — Multiplex PCR amplification of target GAPDH regions. Tumor samples (T1-2, T2-2, T3S1-2, T3S2-2, and T4-2) display failed or lower amplification of larger targets. Corresponding normal samples (N1-2, N2-2, N3-2, N4-2) are shown for comparison. A no template control (NTC) and a positive reference control (R1 (Pos)) lane are shown. (TIF) [file pone.0088163.s001.tif]
